# Laser vs. thermal treatments of green pigment PG36: coincidence and toxicity of processes

**DOI:** 10.1007/s00204-021-03052-w

**Published:** 2021-05-04

**Authors:** Elvira Maria Bauer, Daniele Cecchetti, Ettore Guerriero, Steven Nisticò, Giulia Germinario, Simona Sennato, Lorenzo Gontrani, Pietro Tagliatesta, Marilena Carbone

**Affiliations:** 1 Italian National Research Council, Institute of Structure of Matter (CNR-ISM), Via Salaria km 29.3, 00015 Monterotondo, RM Italy; 2 Department of Chemical Science and Technologies, University of Rome Tor Vergata, Via della Ricerca Scientifica 1, 00133 Rome, Italy; 3 Italian National Research Council, Institute of Atmospheric Pollution Research (CNR-IIA), Via Salaria km 29.3, 00015 Monterotondo, RM Italy; 4 Department of Health Sciences, University of Magna Graecia, Catanzaro, Italy; 5 Department of Pure and Applied Science, University of Urbino, Piazza Rinascimento 6, 61029 Urbino, Italy; 6 Institute of Complex Systems, National Research Council (CNR-ISC), Sapienza Unit, and Physics Department, Sapienza University, P.le A. Moro 5, 00185 Rome, Italy; 7 Department of Chemistry, Sapienza University of Rome, P.le A. Moro 2, 00185 Rome, Italy

**Keywords:** PG36, Pyrolysis, Nd:YAG laser, Additives, Solid residues, Toxic fragments, Harmful morphology

## Abstract

Comparative laser and thermal treatments were carried out on PG36, a green phthalocyanine-based pigment, permitted in European countries where legislation on tattoo composition was issued. Prior to the treatments, PG36 was characterized by SEM imaging, EDX, IR and UV–Vis spectroscopies, revealing an excess of Si and C and O as compared to the pure halogenated Cu-phthalocyanine. Laser treatments were carried out with a Nd:YAG device applied to H_2_O and propan-2-ol dispersions. Pyrolysis and calcinations were carried out in air or under N_2_ flow. The outcome of the different procedures was analyzed by UV–Vis spectroscopy, GC–mass spectrometry, X-ray diffraction of the solid residues, SEM microscopy and dynamic light scattering. The comparative analysis indicated the production of different fragment compounds depending on the treatment, (pyrolysis or laser), and, to some extent, to the solvent of the dispersion, with pyrolysis generating a larger number of hazardous compounds. Hydrocarbons and cyclic siloxanes present as additives in PG36 were stable or degraded depending on the treatment. The morphology of the products is also treatment-dependent with nanoparticles < 20 nm and fibers being produced upon laser treatments only. Based on the experimental findings, the equivalence of laser and thermal treatments is evaluated.

**Supplementary Information:**

The online version contains supplementary material available at 10.1007/s00204-021-03052-w.

## Introduction

The diffusion of the tattoo practice is on the raise worldwide. Up to 2016, most accessible surveys depicted an increasing popularity of tattoos in western countries (Everts 2016; Piccinini et al. 2016). More recently, an online survey, through email interviews, including Russia, China, and Brazil, indicated how tattooing is becoming fashionable also in those countries, though the motivations for tattooing may be different depending on the cultural background (Kluger et al. 2019). Additional studies report how tattooing is increasing in South Korea (Kim 2017) and that South American countries score highest in tattoo-related Google searches in the period 2004–2018 (Kluger 2019). Although the absolute figures of tattooed people may vary with the survey, the increasing trend of tattooing practice is unequivocal and in all cases, a gender swap emerges, with a larger number of tattooed women as compared to men (Kluger et al. 2019 and Dalia Research 2021) and an increase of average age of tattooed people from 18–24 (Laumann and Derick 2006) to 36 years (Dalia Research 2021). The skyrocketing diffusion of tattoos triggered studies on several aspects of tattoo-related issues, ranging from adverse effects such as tattoo induced allergies (mostly caused by red inks) (Serup et al. 2019), to carcinogenicity suspects (Kluger and Koljonen 2012), the impact on daily-life issues such as routine medical tests (MRI—magnetic resonance imaging—for instance) (Alsing et al. 2020) and the use of tattoo inks to elicit interest in chemical education (Donia et al. 2021). In case of afterthoughts and regrets, tattoos may be removed either by laser treatments or by cover up, a procedure that usually works better after a preliminary partial laser bleaching. The practice of tattoo removal, however, carries potential risk due to pigment cleavage into toxic or carcinogenic fragments upon tattoo laser treatment. Experiments carried out so far indicate that potentially hazardous 2-methyl-5-nitroaniline, 4-nitrotoluene, 2,5-dichloroaniline and 1,4-dichlorobenzene may generate upon Nd:YAG laser treatment of monoazo red pigments PR22 (C.I. 12315) and PR9 (C.I. 12460) (Vasold et al. 2004). Hauri and Hohl (2015) performed a systematic study on decomposition products of red pigments by comparing sunlight and laser treatments. In some cases, 2-amino-4-nitrotoluene, 3,3-dichlorobenzidine and o-toluidine were identified as laser decomposition products. In addition, toxic hexachlorobenzene was found as product of the Nd:YAG laser irradiation of pig skin tattooed post-mortem with the yellow quinophthalone PY138 (C.I. 56300). Phenyl isocyanate, benzene, aniline and 3,3′-dichlorobenzidine were found when the pig skin was marked with the orange diazopigment PO13 (C.I. 2.1110) (Hering et al. 2018).

Pyrolysis and laser treatments are sometimes presented as equivalent processes leading to the pigments fragmentation and subsequent removal. In particular, the copper-phthalocyanine blue pigment PB15:3 has been investigated by pyrolysis under the assumption that the laser treatment would be photothermal, based on the lack of fragmentation under UV–visible irradiation (Schreiver et al. 2015). In addition, they found the Ruby laser effective in PB15 fragmentation, at variance with Nd:YAG both at the fundamental and doubled frequencies (1064 and 532 nm). The main decomposition products of PB15:3 upon thermal or laser treatments are benzonitrile, benzodinitrile and HCN. Additional 2-butenone and benzene traces can be seen either as additive or impurities. When dealing with halogen-substituted copper phthalocyanine, the overall fragmentation pattern becomes more complex, because of the halogenated fragments which may generate (Germinario et al. 2015). Furthermore, additives may be present on pigments, for varying the dispersibility properties, which may have a different behavior under thermal or laser treatments. Since all the produced fragments may have a different degree of toxicity, we find it important to determine whether laser or thermal treatments are fully equivalent on a halogenated Cu-phthalocyanine.

Here, we focus on hexabromodecachloro copper phthalocyanine, usually labeled as PG36, a permitted pigment, in the European countries where regulations are issued (Code of Federal Regulations 2015; ResAP 2008; Regulations in single European countries 2008, 2012, 2013a, b, TätoV 2008; Swiss Regulation 2005) though multiple infringements and false declarations were reported by Hauri (2014), and Bauer et al. (2019), indicating often the presence of the restricted PG7 (hexadecachloro copper phthalocyanine), instead of PG36. Prior to any treatment, we carried out a control characterization of the PG36, by IR, SEM, EDX and DLS measurements and determined the elemental composition, the type of aggregation of the phthalocyanines, as well as the average size in propan-2-ol and water dispersions. We, then, compared the effects of thermal and laser treatments of PG36 in connection with the potential presence of additives. The laser treatments were carried out with Nd:YAG operated at 532 nm, pulse duration 2–6 nm, on PG36 dispersed either in propan-2-ol or in water, at different irradiation times. In choosing the solvents, we took into account the different stratification of the skin layers, and their different polarities. More in detail, the residence of the tattoo ink in the skin largely depends on the healing stage and successively on the time elapsed since the tattoo was performed (Lea and Pawlowski 1987; Darvin et al. 2018). In the initial stage, the inks particles are distributed through the epidermis and the dermis, damaged by the needles during the ink injection. Through the healing phases, the epidermal–dermal junction reforms, and the ink tends to localize in the dermis (papillary layer and reticular dermis). The fibroblasts of the dermis would contain the tattoo ink, surrounded by collagen, thus being responsible for the ink life span. The different layers of epidermidis and dermis have different polarities (Persa et al. 2021; Muroyama and Lechler 2012), which also play a role in the epithelial metabolism (Zhuang et al. 2018; Gomes et al. 2019). Throughout this procedure, the additives may be entrapped in the fibroblasts to an extent that depends on how far they are embedded with the pigments, how the polarity of the layers affects their solubility and the efficacy of the phagocytes which intervene in the healing process in sweeping them away. The irradiation products were, then, analyzed by GC–mass spectrometry, UV–Vis spectroscopy, DLS and SEM microscopy. Thermal treatments consisted of pyrolysis at 700 and 800 °C followed by GC–mass analysis. In addition, calcinations of PG36 were carried out at 800 °C in air or under nitrogen flow. The solid residues from pyrolysis were characterized by SEM, and DLS (subsequent dispersions), whereas the calcined samples were also subjected to XRD and IR analyses. We found that the types of fragment compounds are dependent on the treatment, the additives playing a role in determining products. The solid residues of calcination procedures contain CuO and SiO_2_. In addition, CuBr, CuCl, Cu_2_Cl_2_O and possibly Cu_2_O are also obtained for calcination under N_2_. The morphology of the products is treatment dependent as well, with nanoparticles (< 20 nm) and fibers generated by laser treatments only. Based on the experimental findings, the equivalence of laser and thermal treatments and their associated toxicity is evaluated.

## Materials and methods

### Chemicals

The pigment PG36 was purchased from Schminke Künstlerfarben. Propan-2-ol, ethyl acetate, ethanol, DMSO and ultrapure water were purchased from Merck. All chemicals used in this investigation were of reagent grade and used without any further purification.

### Sample preparation

The PG36 powder was dispersed in water or propan-2-ol at a concentration of 0.9 mg/ml, by sonicating at 40 kHz for several hours, until a good dispersion was reached without residual deposits on the bottom of the flask. Care was taken to avoid overheating of the sonication bath, by replacing the water of the bath every 15 min, and avoid thermally induced aggregation of the pigment. The dispersions are, then, diluted at 0.09 mg/ml and further sonicated for 1 h, for the laser treatments.

### Calcination

The calcinations were carried out in muffle oven for 3 h at 800 °C either in air or under nitrogen flow. The calcined in air appears as a brown-greenish powder, the one calcined under nitrogen flow clearly has a blackish color containing also light-red grains. Both powders are shown in Fig. SI1.

### Equipment and data analysis

Gas chromatography–mass spectrometric analyses were carried out with a triple quadrupole gas chromatograph/mass spectrometer (Trace 1310GC/TSQ 8000 Evo, Thermo Scientific, USA) and the chromatographic separation was performed by a DB/XLB column (60 m 0.25 mm, 0.25 I.D., Agilent J&W, USA) with hydrogen as carrier gas at 3 ml min^−1^ flow rate. The injected volume is always 1 μl in splitless mode at 250 °C. The oven program was the following: isothermal at 90 °C for 5 min then increased at 10 °C min^−1^ to 280 °C (hold for 5 min). The MS operating conditions were the following: positive electron ionization (EI +) mode with electron energy of 70 eV and and emission current of 50 μA. Acquisition was made in scan mode 35–450 *m*/*z* in 0.2 s. The transfer line and ion source temperatures were kept at 290 and 300 °C, respectively. GC/MS peak identification was conducted using the software Xcalibur 2.2 by Thermo Fisher Scientific. PG36 in propan-2-ol and water as well as upon laser treatments and calcination procedure have been analyzed by Dynamic Light Scattering (DLS) to get their size and size distribution. A Malvern NanoZetaSizer instrument with HeNe laser and backscattering detection (173°) was employed (Malvern Instruments LTD, UK). This set-up is less sensitive to multiple scattering effects and dust than the commonly used 90° geometry. Measurements have been performed at 298 K by a thermostatted cell holder controlled by a Peltier system. The diffusion coefficients D of the particles obtained by analysis of the DLS autocorrelation function of scattered intensity are converted in a distribution of apparent hydrodynamic radii *R*_H_ using the Stokes–Einstein relationship *R*_H_ = *k*_B_*T*/6пη*D*, where *k*_B_*T* is the thermal energy and η the solvent viscosity. As commonly used for polydisperse samples, we applied the method of cumulants where the logarithm of the first-order DLS autocorrelation function is expanded in power series and from the first and second cumulant coefficients the average hydrodynamic size RH and the polydispersity index (PDI) can be determined (Pecora 2000). This method gives the most direct and robust measure of the size since it does not rely on the details of scattering. Moreover, it is directly obtained from the initial part of the autocorrelation function where the signal-to-noise ratio is largest. When PDI values are larger than 0.2–0.3, two or more populations with distinct size could be present in the dispersion and the analysis has to be carried out with proper methods able to detect the detailed size distribution of the sample. To ascertain this point, we used intensity-weighted NNLS algorithm. Note that the *R*_H_ obtained by this analysis is biased on larger size because the scattered intensity is proportional to the sixth power of the particle size, according to Mie scattering. SEMs were collected with a Zeiss Auriga 405, Field Emission Scanning Electron Microscope instrument, mounting a Gemini column and operating at 7 kV. The EDX analyses were made by coupling the Field Emission Scanning Electron Microscope (SUPRA™ 35, Carl Zeiss SMT, Oberkochen, Germany) with the Energy Dispersive Microanalysis (EDS/EDX, INCAx-sight, Model: 7426, Oxford Instruments, Abingdon, Oxfordshire, UK), operating at 20 kV. XRD patterns of the calcined samples were collected with an X’pert pro X-ray diffractometer by Philips, using CuK-Alpha radiation. They were, then, analyzed by a Rietveld procedure with the GSAS-II suite of programs (Toby and Von Dreele 2013). Infrared spectra were recorded with a Shimadzu Prestige-21 FT-IR instrument, equipped with an attenuated total reflectance (ATR) diamond crystal (Specac Golden Gate), in the range 400–4000 cm^−1^, with a resolution of 4 cm^−1^. The UV–Vis spectra were recorded with a Perkin Elmer, Lambda 950 spectrophotometer. Laser treatments were performed with a Deka Duolite QS Laser operated at 532 nm at a fluence of 3.5 J/cm^2^ and a spot size of 4 mm. Additional low fluence treatments were performed for morphological comparison purposes at 0.21 J/cm^2^. The pyrolysis experiments were carried out with a microfurnace pyrolyser injection system Pyrojector II (SGE, USA). Small amounts (< 0.1 mg) of sample were inserted into a quartz tube (4 cm × 0.53 mm) which was then introduced into the microfurnace operating at two pyrolysis temperatures (700 and 800 °C) and at a pressure of 15.0 psi. The pyrolysis chamber was directly connected to the injection port of a Clarus 680 (Perkin Elmer) gas chromatograph coupled to a Clarus SQ 8T (Perkin Elmer) single quadrupole mass spectrometer. An Elite-5MS (30 m × 0.25 mm i.d., 0.25 µm film thickness) was used with the following GC oven program: 40(4)-15-280(10). Helium was used as carrier gas at a constant pressure of 10.0 psi. Injector, transfer line and ion source temperatures were set at 280, 290 and 250 °C, respectively. Mass spectra were acquired in the positive ionization mode (70 eV) in a mass range of 45–450 *m*/*z*. Solvent delay was set at 0.5 min. and the analyses were performed in split mode (1:50). In Table 1, a summary of the PG36 treatments and corresponding naming of the samples is reported.

**Table 1:** Summary of the conditions used for PG36 laser and thermal treatments, and corresponding samples naming

Sample	Treatment	Solvent/carrier	Fluence (J/cm^2^)	Spot size (mm)	Temp (°C)	Time (min)
P2OL15	Nd:YAG	Propan-2-ol	3.5	4		15
P2OL44	Nd:YAG	Propan-2-ol	3.5	4		44
H_2_O15	Nd:YAG	H_2_O	3.5	4		15
H_2_O44	Nd:YAG	H_2_O	3.5	4		44
Py700	Pyrolysis	He			700	
Py800	Pyrolysis	He			800	
CA800	Calcination	Air			800	180
CN800R	Calcination	N_2_			800	180
CN800B	Calcination	N_2_			800	180

CN800R and CN800B were obtained upon the same calcination procedure, followed by coarse manual separation, based on the color

## Results and discussion

### Pigment characterization

**Fig. 1:**
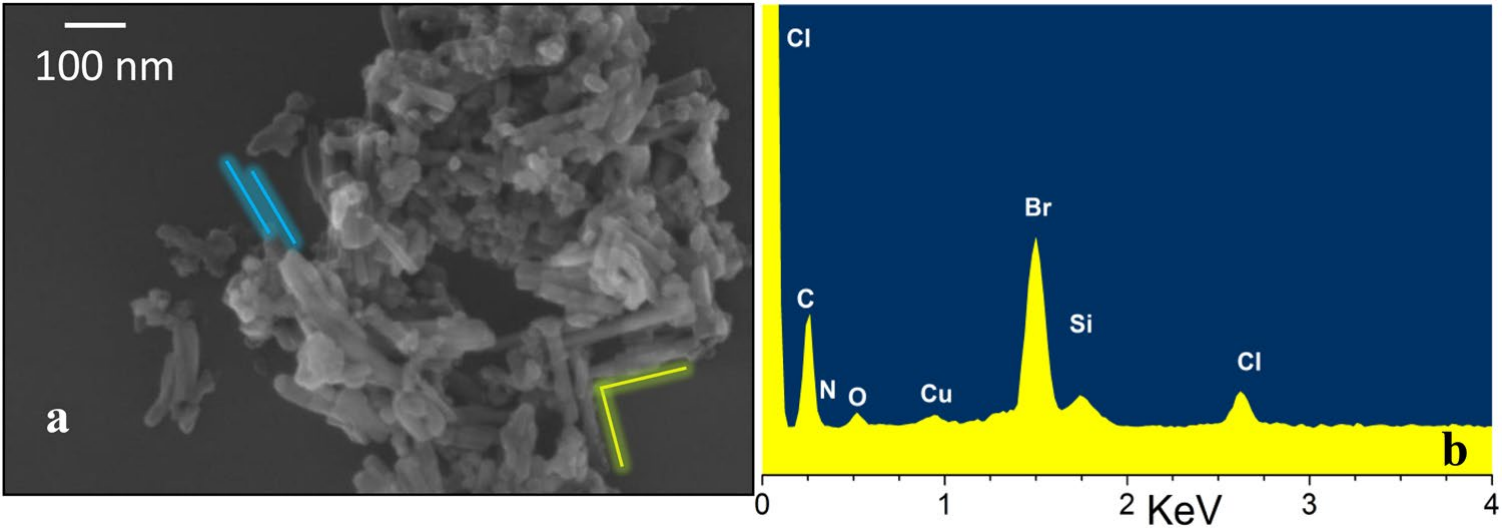
**a** SEM image of PG36. The *blue* and *yellow lines* are examples of parallel and nearly perpendicular macro-arrangements of the aggregates. **b** EDX analysis after deposition on carbon tape, hence excluding Si contribution from the sample holder (color figure online)

PG36 was characterized through several techniques. In Fig. 1, a SEM image and the EDX analysis are reported.

The SEM image of the PG36 presents aggregates of rod-like shape, with the longest dimension in the 50–300 nm range, and the smallest one in the 15–45 nm range. Parallel and nearly perpendicular rows as in alpha and beta arrangements are highlighted in the figure, by the blue and yellow bars, respectively. Additional roundish aggregates can be observed with diameters between 15 and 30 nm (Fig. 1). In a similar commercial product, the PG7 presents mostly roundish aggregates with average diameter of 70 nm (Fig. 2 of Bucella et al. 2018). The binding of substituents on meso positions favors the rod-like aggregation (Wang et al 2014). The EDX analysis of PG36 was reported in Bauer et al. (2020). However, we repeated it here using a different sample-holding construction, to avoid external Si contribution (Fig. 1a, Table 2). More in detail, PG36 was deposited on a layer of carbon tape on top of the Al_2_O_3_ stub, which is then mounted on the carousel for the analysis. This way, we could verify the presence of 1.4 atomic % of Si in PG36 and confirm the higher ratio of Br:Cl atomic %, with respect to the nominal one. The C content is distorted by the use of the carbon tape, but, in the previous analysis, it also appeared excessive, as compared to a pure halogenated Cu-phthalocyanine.

**Fig. 2:**
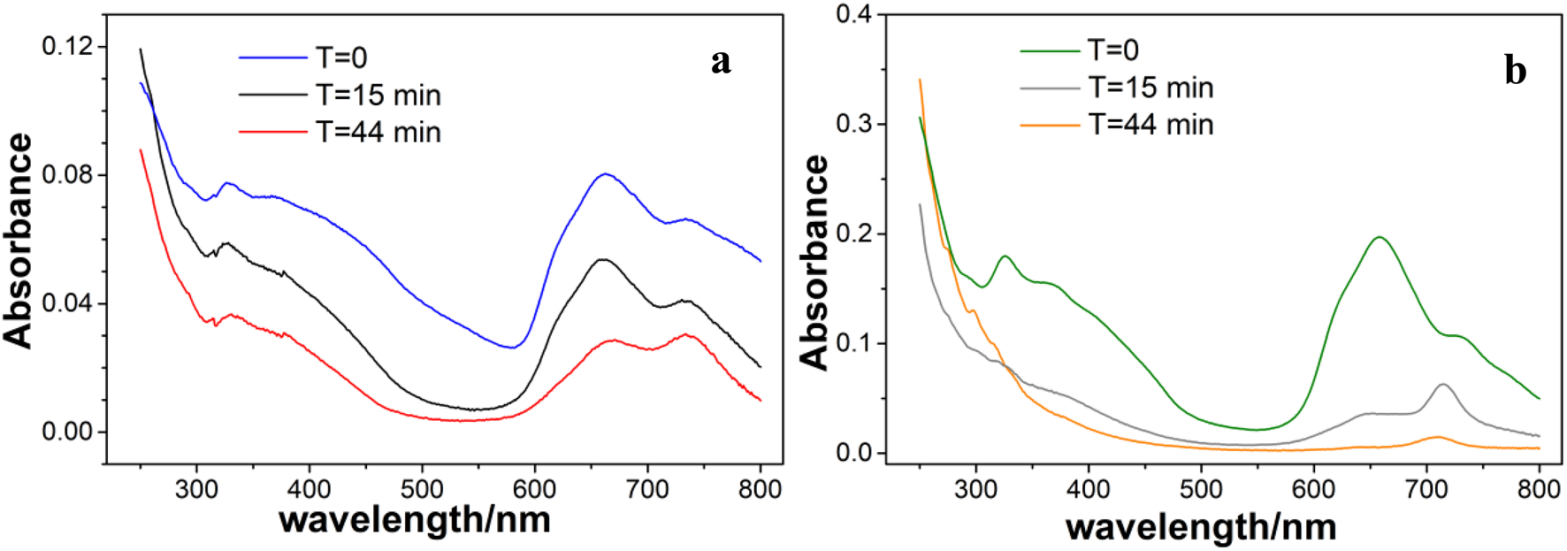
UV–Vis spectra of PG36 treated with Nd:YAG laser operated at 532 nm, at different irradiation times, **a** dispersed in water and **b** dispersed in propan-2-ol. The spectra are compared to the untreated dispersions (*T* = 0) (color figure online)

**Table 2:** EDX elemental analysis of PG36 deposited on carbon tape (C-SH) or Si (Si-SH) sample holders

Element	PG36 atomic % C-SH	PG36 atomic % C-SH norm	PG36 atomic % Si-SH
C	71.7 + 17.04	88.58	83.3
N	2.01	2.1	2.5
O	2.39	2.39	7.7
Br	3.99	4.01	4.8
Cl	1.24	1.27	1.4
Cu	0.23	0.25	0.3
Si	1.40	1.4	

The atomic %s are normalyzed for the contribution of the sample holder, i.e., the atomic % of Si or Si + F (PG36)

The DLS measurements of PG36 were carried out in water and propan-2-ol, since they are the solvents used afterwards for the laser treatments. In general, these two solvents were chosen for their different polarity, which mimics, to some extent, the different polarity of the various skin layers. Though the two methods of analysis show a little systematic discrepancy in the obtained value of size, they both point at a single population of the particles in both solvents. More in detail, PG36 has an average hydrodynamic size of 98 nm in water and 126 nm in propan-2-ol, according to cumulant analysis, and slightly larger values of 131 and 155 nm, respectively, when size distribution analysis by NNLS algorithm is employed (Table 3). The powder analysis by SEM shows mostly asymmetric aggregates, i.e., elongated ones, with a large size distribution, especially in the long direction and a few roundish particles. The single distribution population indicates a rearrangement of the aggregation in both solvents, to become more uniform, though, on average, they are slightly larger in propan-2-ol, as compared to water.

**Table 3:** Average hydrodynamic diameter 2*R*_H_ from DLS measurements on PG36 in H_2_O (PG36/H_2_O), and PG36 in propan-2-ol (PG36/P2OL), determined by cumulant analysis and intensity-weighted size distribution by NNLS algorithm

Sample	2*R*_H_ (nm) (cumulant)	PDI (cumulant)	2*R*_H_—Peak 1 (nm) (NNLS)
PG36/H_2_O	98 ± 1.2	0.259 ± 0.012	131 ± 4.2
PG36/P2OL	126 ± 1.6	0.265 ± 0.025	155 ± 3.2

Data refer to average value and standard deviation over three repeated measurements

The IR spectrum was taken directly on the powder and is reported in Fig. SI2. The corresponding assignments were previously reported (Bauer et al. 2019, 2020). Briefly, the IR spectrum shows the aromatic C=C, and C–N stretching and C–N–C in-plane bending of the macrocyclic phthalocyanine, in the spectral region 1600–1500 cm^−1^. The region 1400–1000 cm^−1^ is characteristic of completely substituted copper phthalocyanine derivatives because of the C–H-related missing features. The medium to strong bands at 775–765 cm^−1^ are tentatively assigned to the C–Cl stretching vibrations, whereas the broadening of the band around 745 cm^−1^ is ascribed to C–Br bonds. Inspection of the UV–vis spectrum of pigment PG36 dissolved in DMSO revealed an evident red-shift of the Soret and Q bands when compared to an un-substituted Cu-phthalocyanine and a fully chlorinated one (not-shown here).

The UV–Vis spectra of PG36 dispersed in water and propan-2-ol are reported in Fig. 2a,b) (*T* = 0, untreated samples). They both present the typical B and Q bands of Cu-phthalocyanine, with peaks at 326, 370, 662, 732 nm and a shoulder at 622 nm, for the dispersion in water and slightly shifted peaks at 326, 368, 658, 735 nm and a shoulder at 618 nm for the dispersion in propan-2-ol. It must be noted, however, that the absorption of PG36 in propan-2-ol is larger as compared to water. The intensity of the two spectra is remarkably different as it can be deduced from the scale of the absorption spectra or estimated as height of the peak at 662 nm (658 nm for propan-2-ol) with the respect to the bottom level of the baseline at 575 nm, which is roughly 3:1 higher in propan-2-ol. Since both dispersions have nominally the same concentrations, and similar extinction coefficients in the monomeric form (Kihara et al. 2019), we conclude that PG36 disperses better in propan-2-ol, than in water. It must be added that the absorption intensity of phthalocyanines can be influenced by the particle sizes (Kihara et al. 2019), but the size difference of PG36 in the two dispersions is in the order of 20%, hence cannot account for the different absorbance.

PG36 was subjected to laser and pyrolysis treatments and the outcomes probed with different techniques. Furthermore, calcination of PG36 were carried out under air or nitrogen and analyzed by XRD.

### PG36 laser treatments and pyrolysis

#### UV–Vis spectra after laser treatments

The dispersion of PG36 upon laser treatments were, then, analyzed by UV–Visible spectroscopy. The spectra are reported in Fig. 2a, b, for water and propan-2-ol dispersions, respectively, for different irradiation time (*T* = 15 min and *T* = 44 min) and compared to the untreated samples (*T* = 0).

As far as the dispersion in water is concerned, the laser irradiation has an overall effect of intensity decrease, indicating a partial fragmentation of the Cu-phthalocyanine into moieties non-absorbing in the UV–Vis range. In addition, a swap of intensity is observed between the peaks at 662 and 732 nm, with the latter peak becoming higher than the former upon irradiation. This can be correlated with a transition from alpha to beta phase which is known to be promoted by heating thin solid Cu-phthalocyanine (Lucia and Verderame 1968) as well as Cu-phthalocyanine nanowires (Tong et al. 2007). The alpha-to-beta conversion was considered part of the growing mechanism of hexadecafluoro Cu-phthalocyanines nanowires, induced by Nd:YAG laser irradiation and subsequent seeding (Kihara et al. 2019).

A new feature emerges from the UV–Vis spectra of laser-treated PG36 dispersed in propan-2-ol at 715 nm. A similar feature was identified at 708 nm in the UV–Vis spectra of the laser-treated Green Concentrate ink, based on PG7 and attributed to the Cu(II) octachlorophthalocyaninate (Bauer et al. 2020). The shift from 708 to 715 nm in the current spectra may be related to a mixed Cl_*x*_Br_8-*x*_ phthalocyaninate, where some of the chlorine atoms have been replaced by bromine ones. As in the previous case, it cannot be assessed whether this is a by-product associated to the PG36 pigment or a product of the PG36 decomposition. Regardless of its origin, no information is available on the associated toxicity, whereas Cu (II) octachlorophthalocyaninate is reported as inactive by EPA (TSCA Inventory 2021).

The intensity of the spectra of PG36 dispersions in water and in propan-2-ol upon irradiation is comparable, whereas at *T* = 0, the initial dispersion in propan-2-ol yields a spectrum three times more intense, than in water. This indicates more efficient decoloring processes (including decomposition) in the former solvent. Furthermore, the features associated with the octahalogenphthalocyaninate emerge in propan-2-ol only, thus indicating either that the compound is better evidenced by the larger intensity reduction or that it is generated in this solvent only.

### Laser treatments and pyrolysis: GC–mass spectrometry

Laser treatments and pyrolysis give rise to PG36 fragmentations, whose patterns are complex and medium dependent. In general, we singled out three types of compounds, halogenated fragments, hydrocarbons and siloxanes.

The list of fragments produced is reported in Table 4 for the samples H_2_044, P2OL44 and Py800.

**Table 4:**
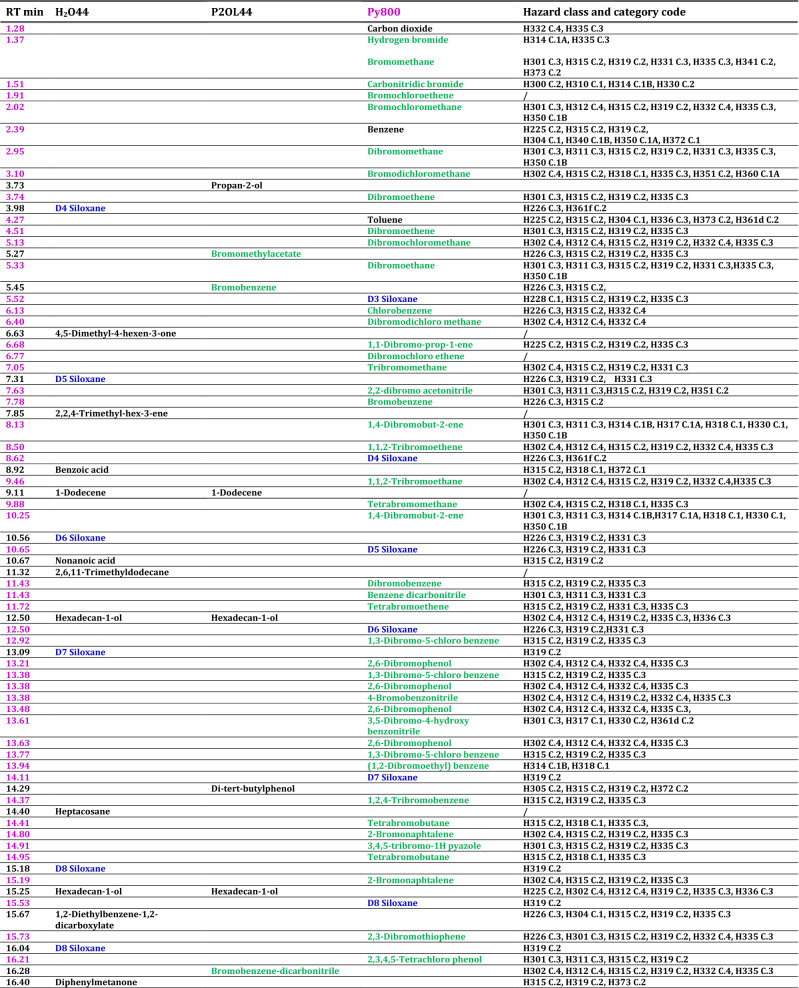

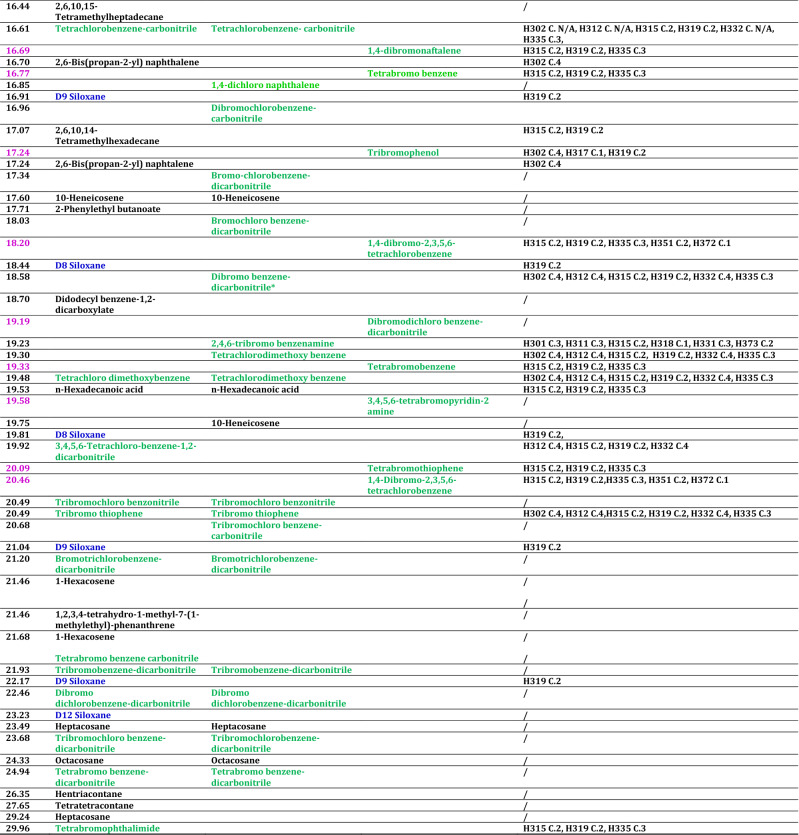
Fragment compounds produced upon pyrolysis and laser treatments along with the corresponding hazard codes and categories

The retention times (RT) are marked lilac for fragments due to pyrolysis, black for laser. Halogenated fragment compounds are reported green, siloxane blue and hydrocarbon black. The hazard codes are labeled H followed by a three digits number, the category is indicated as C. followed by a number ranging from 1 (highest risk) to 4 (lower risk). The slash “/” indicates that hazard data are not available for the corresponding compound. Finally, siloxanes are indicated by the labels D (cyclic siloxanes) followed by a number corresponding to silicon atoms in the structure. The complete formulas are Hexamethyl cyclotrisiloxane (D3), Octamethyl cyclotetrasiloxane (D4), Decamethyl cyclopentasiloxane (D5), Dodecamethyl cyclohexasiloxane (D6), Tetradecamethyl cycloheptasiloxane (D7), Hexadecamethyl cyclooctasiloxane (D8), Octadecamethyl-cyclononasiloxane (D9), Tetracosamethyl-cyclododecasiloxane (D12)

In Table SI1, the main mass losses of the fragment compounds produced either by pyrolysis or by laser treatments are reported. The GC–mass spectra of the samples H_2_015, P2OL15 and Py700 are characterized by a lower number of fragments, and/or by peaks at lower intensity as compared to the corresponding samples treated longer (H_2_044, P2OL44) or at a higher temperature (Py800) and they are not reported. In Tables 4 and SI1, the halogenated fragments have been marked in green, the hydrocarbons black and the siloxanes blue. The halogenated fragments produced in water are all (but one) benzene molecules substituted in all 6 positions, the exception being a fivefold substituted benzene (tetrabromobenzonitrile). In the sixfold substituted benzene, 4 substituents are halogens, either of the same type (all Br or all Cl) or they are Br and Cl in ratio 3:1 or 1:3. The other two positions carry two nitriles or, in one case, two methoxy groups (tetrachlorodimethoxybenzene). The fragmentation pattern of PG36 in propan-2-ol is more complex and reveals the additional presence of mono-substituted benzene (bromobenzene) as well as tri-, and tetra- substituted benzene derivatives with one or two nitriles attached, bromoethylacetate, 1,4-dichloronaphthalene and 2,4,6-tribromobenzenamine. The pyrolysis of PG36 at 800 °C produces a far more complex envelope of halogenated fragments, which include brominated and chlorinated aliphatic hydrocarbons of methane, ethane, ethene, propene, butane and butene, along with dibromonitrile, and mono-, di-, tri-, tetra-, penta-, and hexa-substituted benzene derivatives. All treated sample also reveal the presence of bromine-substituted thiophene. Furthermore, HBr is detected as treatment product in Py800.

The differences between laser and thermal treatments of PG36 extend also to the other types of fragments, since siloxanes are detected upon laser treatments in water, and thermal treatment, but not after laser treatments in propan-2-ol. The same holds for hydrocarbons, which are detected upon laser treatments in both solvents (though better in water, i.e., with more types of different hydrocarbons detected, than in propan-2-ol), but not after the pyrolysis (with the exception of aromatic compounds related to benzene and toluene).

The hydrocarbons detected after laser treatments are long linear chains of alkanes, such as tetratetracontane, of alkenes (10-heneicosene), terminal alcohols (hexadecan-1-ol), or terminal carboxylic acids (*n*-hexadecanoic acid). In addition, branched hydrocarbons (e.g., 2,6,10,14-tetramethylhexadecane) and aromatic molecules (e.g., benzoic acid, di-*tert*-butylphenol) are present as well as a long chained substituted aromatic molecules (didodecyl benzene-1,2-dicarboxylate). It is noteworthy that branched and aromatic hydrocarbons survive the laser treatment in water, whereas almost only linear chain hydrocarbons remain upon laser treatment in propan-2-ol.

The pH of the PG36 water dispersions was checked before and after laser treatments and did not display any variation, thus excluding a significant production of HBr, possibly undetected by GC–mass spectrometry.

For comparison purposes, GC–mass spectra were taken of water and propan-2-ol supernatant of PG36 prior to the laser treatments. The outcome is reported in the Table SI3 of the Supplementary Information. In particular, the compounds present both before and after the laser treatments are reported in light blue, whereas few additional molecules which are detected only in the supernatant are marked violet. However, these compounds are either isomers of molecules detected upon treatment, or hydrocarbons which the skeletal chain one methyl unit longer than after the treatment (i.e., decanoic vs nonanoic acid), or hydrocarbons, such as tetradecane and nanodecane, whose skeletal chain is on average shorter than the hydrocarbons which emerge upon laser treatments. In general, high molecular weight hydrocarbons are not detected in the supernatant. Siloxanes are present in the water supernatant only. The detection of high molecular weight hydrocarbons only upon laser treatment supports the adherence between pigments and additives, which are freed only upon breakage of the pigment.

The presence of hydrocarbons and siloxanes can be related to PG36 treatments during the production phase. Pigments are typically subject to surface treatments, coating and encapsulation during synthesis or post-synthesis aiming at inhibiting crystal growth (Fisher 1950), decreasing powder cohesion and enhancing pigment dispersibility (Schröder 1988) and providing higher affinity with selected interfaces, such liquids or for binders (Bieleman 2000). Among pigments, Cu-phthalocyanine-based ones are considered among the most treated ones (Salis-Gomes et al. 2019). Silicon dioxide, siloxanes (polymers or as oligomers) and organo-functional silane are listed among possible coatings (Bugnon 1995), which can also act as antifoaming agents. Furthermore, long chain carboxylic acids (fatty acids) (Swarup and Schoff 1993) and poly (12)-hydroxystearic acid (Vernardakis 2007) are common surfactants used during the pigments’ synthesis. In addition, as far as Cu-phthalocyanine pigments are concerned (usually blue ones), they are treated by milling the crude with a pigment derivative such as sulfonated phthalimidomethyl phthalocyanine (Vernardakis 2007). The latter process, in particular, may account for the detection of tetrabromophthalimide in the H_2_044 sample. In pyrolyzed samples, the presence of thiophene derivatives (brominated) is evidenced. Sulfur can be found in phthalocyanine-based pigments as the outcome of a sulphonation process, aimed at improving the dispersibility (Salis-Gomes et al. 2019). In these regards, it must be noted that the pyrolysis experiments were performed on pigments PG7 and PG36 from a different supplier (Kremer Pigmente GmbH) (Fardi et al. 2018; Germinario et al. 2015) which can account for such a treatment. Thiophene, on the other hand, belongs to the mixture of hydrocarbons added to the pigments rather than being the outcome of a derivatization process.

The presence of hydrocarbons and siloxanes may affect the removal and the type products upon different treatments. On the other hand, once the pigments are injected under the skin, the adherence of the additives, and their limited solubility, depending on the skin layer, support their residence in the skin, along with the pigments. The overall fragmentation pattern is, in general, quite complex. However, a few rationalizations are possible, which also aid the understanding of the role of pigments and additives in the whole processes:

1. The most abundant production of different halogenated fragments is observed for the pyrolized sample. The process occurs under He flow, hence in the absence of air, but in the presence of hydrocarbons and with production of CO_2_.
2. Propan-2-ol does not retain siloxanes upon laser treatment. Since they are present as additives with PG36, we must conclude that they agglomerate to an extent that prevents them to be detected by GC–mass spectrometry, possibly to form SiO_2_. Siloxane removal from the dispersion may be correlated with the efficiency of discoloration and decrease of the UV–Vis intensity as compared to water dispersions.
3. The laser treatment of PG36 dispersed in water retains siloxanes, hydrocarbons and produces only large halogenated fragments. Overall, this is the least efficient process in terms of fragments production, which is characterized by a moderate discoloration of the dispersion, and where part of the irradiation energy is converted into energy for a phase transition (*alpha*-to*-beta*), rather than for fragmentation of pigment and additives.

Overall, the significantly different fragmentation pattern in the case of the pyrolysis with respect to the laser treatments may be partly related to the environment, since it is a thermal process in the presence of an inert carrier (He gas), and of additives, and can be considered the equivalent to an anaerobius process in living organisms or in cell compartments. The heating process in absence of oxygen (from air, or from the solvent), but in presence of the hydrocarbons can be envisaged as a combustion and concomitant halogenation (mostly bromination), which would explain the production of small molecules such as methane to butane brominated derivatives, as well as CO_2_. In this framework, this is not sufficient for a siloxane removal, which remains in the mixture of products.

The toxicity of the treatments products was assessed by querying the ECHA (European Chemical Agency) database which reports the hazards according to the Classification Labelling and Packaging (CLP) regulation, aligned with the Global Harmonized System (CLP Database 2009). If not available in the primary database, toxicity information was from the CHEM SPACE-associated database (CHEM SPACE). However, for a large number of bromine-substituted molecules, as well as for some of the hydrocarbons, and some siloxanes, the toxicity information was not available, thus effectively undermining the possibility of achieving a complete harmfulness overview, at variance with PG7 (Bauer et al. 2020), though the latter is a restricted pigment in countries were tattoo inks legislation is issued. The risk codes available associated to the various fragments range from flammability, to eye and skin damage and irritation (H314, H315, H318, H319) or cause allergic reaction of the skin (H317). Some fragments are harmful, toxic or fatal if swallowed and enter the airways (H302, H301, H300, H304), or if in contact with the skin (H312, H311, H310). Even worse, some of the treatments products may cause genetic defects (H340) or are suspected to do so (H341), may cause (H350) or are suspected to cause cancer (H351) may damage (H360) or are suspected to damage the fertility and the unborn child (H361f, H361d) or damage organs upon prolonged exposure (H372, H373). The correspondence between risk code and hazard is reported in Table SI2 of the Supplementary Information. However, since different treatments yield different types of products, with associated hazards, the overall toxicity becomes treatment dependent. In particular, only the pyrolysis of PG36 generates halogenated (brominated or brominated and chlorinated) compounds classified under the risk code carcinogenic, or suspected carcinogenic, i.e., bromochloromethane, dibromomethane, dibromoethane, 1,4-dibromobut-2-ene, 2,2-dibromo acetonitrile, 1,4-dibromo-2,3,5,6-tetrachlorobenzene, bromodichloromethane, with the addition of benzene. A fair comparison between processes, however, is not possible, due to the lack of toxicity information for the large part of halogenated fragments generated by laser treatments in water and in propan-2-ol (such as dibromochlorobenzene-carbonitrile, bromo-chlorobenzene-dicarbonitrile or tribromochloro benzonitrile, for instance). As far as siloxanes are concerned, though generally considered harmless, doubts were casted on potential toxicity in their routine use, especially for the low weight one (Mojsiewicz-Pieńkowska et al. 2016), and indeed some of them currently appear in the ECHA and CHEM SPACE database as potentially toxic. These additives survive as cyclic siloxanes both after pyrolysis and after laser treatments in water dispersion, though with slight differences in the two cases. Since the toxicity of siloxanes is largely related to the cyclic rather than the linear forms, special care was taken in the analysis of the pattern of the mass losses. All GC–mass spectra are checked against the blanks which allow us to exclude possible spurious contributions from column bleeding. Cyclic D4–D8 siloxanes are found in laser-treated water dispersion, whereas D3–D8 are found upon pyrolysis. These siloxanes mostly cause eye irritation (H319, D3, D5–D8), they are flammable (D3–D6), or cause skin irritation (D3). However, D5 and D6 are toxic if inhaled (H331) and D4 is suspected of damaging the fertility (H361f) (OEHHA 2008). Higher siloxanes such as D9 and D12 are likely present too based on the mass losses patterns. However, the mass analysis is limited to a value, which does not include the parent peak of D9 and D12, hence we deem these assignments as solely probable. As far as hydrocarbons are concerned, almost half of them are not classified in the inquired hazard databases. The remaining ones are classified harmful in contact with the skin, the eye and cause respiratory irritation. They are not reported carcinogenic (with the exception of benzene) and in general most hazard is associated with the aromatic molecules which can cause damage to organs or can be fatal if swallowed (toluene).

### Solid residue: IR and XRD

GC–mass analyses upon laser treatments and pyrolysis do not account for residual Cu-containing phases, though Cu is present in the phthalocyanines. On the other hand, the solid residue after the treatments is scarce and/or dispersed in the solvent as well. To have an insight of the solid products of the treatments, we proceeded with the calcination of PG36 at 800 °C (same as the pyrolysis) either in air (samples CA800) or under N_2_ flow (inert as the He flow, sample CN800).

The calcined samples were subjected to IR spectroscopy and the resulting spectrum is reported in Fig. SI3**.** The spectrums of the three calcined samples (CA800, CN800R and CN800B) resulted to be nearly featureless, thus confirming decomposition of pigment PG36 during the thermal treatment. Only several broad and weak vibration peaks can be observed in the 1200–1000 cm^−1^ region. In detail, all calcined samples show a slightly more intense peak at 1090 cm^−1^ accompanied by a shoulder (~ 1120 cm^−1^) and a less intense peak located at 1180 cm^−1^. The peak positions observed can be characteristic for several functional groups containing C–H, C–C or C–O bonds but the presence of siloxanes evidenced in previous experiments has also to be considered. Generally, Si–O–Si bonds are characterized by three different vibrational modes, transverse optical (TO) rocking (~ 460 cm^−1^), TO symmetric stretching (~ 800 cm^−1^) and TO asymmetric stretching (~ 1080 cm^−1^) which is always accompanied by a shoulder. Especially the latter vibrational mode can be tentatively assigned to the peak located around 1090 cm^−1^ and observed in all three samples (Innocenzi 2003). The region below 1000 cm^−1^ is dominated in all three FT-IR spectrums by large and intense bands most properly due to the presence of copper-based residues ruling out further peak identification.

**Fig. 3:**
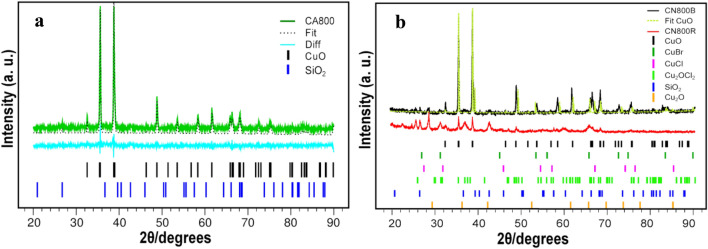
XRD patterns of PG36 **a** calcined in air (CA800) and **b** under nitrogen flow (CN800B and CN800R). In panel **a,** the *green solid line* corresponds to the experimental data, the *black dotted line* is the Rietveld fit, whereas the *light blue solid line* is the fit residual. The reflexes of the phases evidenced in the fit are reported underneath as vertical bars: *black* CuO, *blue* SiO_2_. In panel **b,** the *black solid line* corresponds to the experimental data of CN800B, the *red solid line* to CN800R, the *yellow dotted line* corresponds to the fit with solely CuO. The reflexes evidences in the data analysis are reported as vertical bars; *black* CuO, *blue* SiO_2_, *violet* CuBr, *light blue* CuCl, *green* Cu_2_OCl_2_, *orange* Cu_2_O (color figure online)

XRD spectra were taken of the calcined samples, subsequently analyzed by Rietveld procedure. Measured and fitted XRD spectra are reported in Fig. 3a, b. An expanded version of Fig. 3b is reported in the Supplementary Information for better evidencing the phases detected through the analysis (Fig. SI4). The Rietveld refinement of the structures was addressed, starting from the CA800, since it presents fewer, more intense and narrower peaks than CN800, and by taking into account that residual ash of palladium phthalocyanine combustion in a thermogravimetry experiment was composed exclusively of PdO, based on the mass loss (Lokesh and Adriaens 2013). Analogously, we initially considered that the solid residue would contain copper oxide CuO, only. The crystal structure of the mineral tenorite (100% CuO) was taken from data by Wyckoff (1963), and the simulated powder pattern was built, for Cu Kα wavelength (1.54 Å), modeling its peak shapes with a convolution of pseudo-Voigt functions modified to incorporate asymmetry (Finger et al. 1994). The background was fit with a 6-term Chebyshev polynomial of the first kind, while the unit cell parameters and the instrumental broadening parameters U, V, W were refined as well. Overall, the Rietveld refinement employed 3500 variables and reached the weighted-profile R factor w*R* = 6.62%, *R* = 5.02%, χ^2^ = 7962.43 at convergence. The most evident pitfall of this first refinement is the absence of any peak around 27 degrees 2θ, whereas a small but visible peak is observed in the experimental pattern. After a thorough search, the quartz crystal structure was found to exhibit such feature and consequently SiO_2_ (Antao et al 2008) was added as crystal phase to the mixture in the second cycle of refinement. Upon SiO_2_ addition, the agreement indices improved to w*R* = 6.50%, *R* = 4.95% and χ^2^ = 7677. The optimized composition of the binary mixture was 95.91% tenorite, 4.09% SiO_2_; the calculated pattern is shown as a dotted black line in Fig. 3a. To account for the observed brown-greenish color of the calcined sample, as opposite to the gray to black color of mineral tenorite, even when blended with colorless SiO_2_, a new phase containing tenorite contaminated by other green copper minerals (CuCl_2_ and CuBr_2_) was hypothesised and submitted to the refinement. Yet, very little modification of the tenorite-only pattern was obtained, and CuCl_2_ and CuBr_2_ optimized populations were smaller than 1%. CuO may be coloured in hues from yellow to perceived orange to green when obtained in the nanometer scale. More in detail, it is yellow when CuO particles are in the 2–100 nm range (Sadhvi et al. 2014). The color is light blue to blue, due to a strong phonon–electron coupling when its size diminishes to 1 nm (Tamaki et al. 2014), but it is perceived orange when irradiated at 490 nm, and it is green when it is obtained in homogeneous particles of 40 nm diameter (Vella Durai et al. 2019). The blackish and light-red grains of CN800 were coarsely separated and labeled CN800B and CN800R, respectively. XRD spectra were taken on each batch, separately. The blackish grains showed again a large prevalence of tenorite crystal phase, with only traces of silica and copper halogenides (Fig. 3b, dark gray line), while the light-red portion yielded a quite complicated XRD pattern (Fig. 3b, red line). Though the peaks characteristic of CuO (32.7, 36,6, 38.9, 66.2) are still clearly visible, their intensity is largely smaller. The peaks ascribable to silica are relatively higher, and new peaks of comparable height appear. Noteworthy are the peaks falling at 31.5 and 65.8 degrees 2θ, that can be related to the presence of CuBr (Wyckoff 1963), and those at 26.2 and 37.1 that could be reasonably traced back to an oxychloride Cu_2_OCl_2_ phase (mineral melanothallite, Krivovichev et al. 2002), while CuCl would give a series of peaks (e.g., nantokite powder spectrum (Wyckoff 1963), that are not clearly observed. Furthermore, the existence of copper (I) species that could be produced at high temperature in reduced-air environment, is also evidenced by the likely presence of Cu_2_O in the mixture, in compliance with the reddish color observed. The XRD spectrum of this compound has peaks at 29.6, 36.4, 42.3 and 65.5 (Kirfel and Heichhorn 1990) that would fit nicely with parts of the observed CN800R pattern in that region. Though, a certain degree of overlap is possible with silica peaks at 36.5 and 42.5, and with CuBr one at 65.5 and consequently, Cu_2_O presence cannot be assessed or ruled out by this method without uncertainty. All the analysis of CN800R is admittedly rather qualitative, owing to the overlap of several crystalline peaks and broader signals, probably due to amorphous structures, but it seemingly suggests that mixed halogenated compounds and traces of Cu_2_O can be found in the sample.

The calcination atmosphere has a pivotal role in terms of toxicity, since CuBr, CuCl and possibly Cu_2_O are produced in absence of air oxygen, only. The associated hazards indicate possible skin irritation, eye damage and respiratory irritation (Table 5). No toxicity data are available in ECHA and CHEMSPACE databases on Cu_2_OCl_2._ The safety of CuO and SiO_2_, present in all solid residues, is a complex issue. CuO is considered largely harmless, one of the sources of Cu daily intake (Aguilar et al. 2009), and extensively used for its biocide properties (Carbone 2016; Carbone et al. 2017). Nonetheless, inhalation of high doses of CuO (1.6 mg/Cu aerosol m^3^ for 1 h) resulted in impaired pulmonary functions in rats, the biochemical toxicity of copper in humans occurring when it exceeds homeostatic control (PUBCHEM 2021). Safety issues on SiO_2_ depend on a number of parameters. Nanometric SiO_2_ can cause acute toxicity depending on geometry, porosity, surface characteristics and dose (Yu et al. 2012), whereas the toxicity of SiO_2_ fibers is known, especially with an aspect ratio *L*/*D* > 3 (*L* = length, *D* = diameter) (Boulanger et al. 2014).

**Table 5:** Hazards associated with the solid residues in the calcined samples CA800, CN800B CN800R

X-ray phase	Risk code
CuCl	H302 Cat. 4 Harmful if swallowed
CuBr	H302 Cat. 4 Harmful if swallowed H315 Cat. 2 Causes skin irritation H318 Cat. 1 Causes serious eye damage H335 Cat. 3 May cause respiratory irritation
Cu_2_O	H302 Cat. 4 Harmful if swallowed H318 Cat. 1 Causes serious eye damage H332 Cat. 4 Harmful if inhaled
Cu_2_OCl_2_	/
CuO	Dose dependent
SiO_2_	Dose and shape dependent

### SEM and DLS

**Fig. 4:**
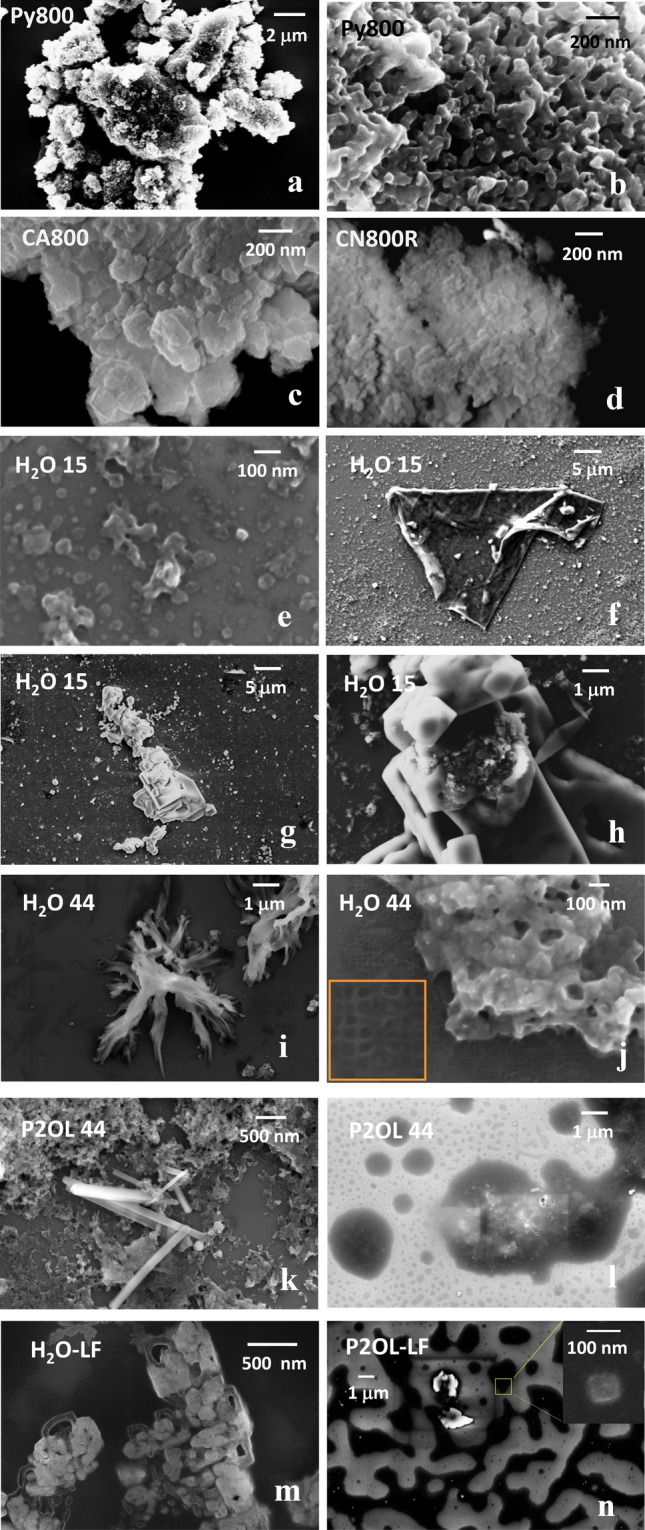
Selected SEM images of laser and thermally treated PG36 in water and propan-2-ol dispersion: **a** is Py800 dispersed in propan-2-ol; **b** is a magnification of **a**; **c** is calcined sample in air and **d** under N_2_, both dispersed in propan-2-ol; **e** through **j** are PG36 samples dispersed in water and laser treated at different times (indicated in the corresponded panel); **k** and **l** are PG36 samples dispersed in propan-2-ol and laser treated. Low fluence (LF) laser-treated samples are reported in panels **m** for water dispersion and **n** for propan-2-ol dispersion (color figure online)

The differences between laser and thermal treatments of PG36 extend to the morphology of the products, with additional distinctions to be made among pyrolyzed and calcined samples. The thermal-treated samples present, in general, large aggregates in the micrometric range, whereas particles in the nanometric range are almost completely absent. The aggregates of the pyrolyzed samples are made of a tri-dimensional network of melted rods which create a porous structure on the surface (Fig. 4a, b sample Py800). Calcined samples lose the tri-dimensional network to a piled-slabs morphology, with a polygonal shape compatible with a CuO component (which usually crystallizes in hexagonal packing) (Fig. 4c, CA800). The size of the slabs is significantly reduced in CN800R as compared probably as an effect of the additional mixed phases (CuBr, etc.), though still keeping the micrometric size of the overall aggregates (Fig. 4d). The scenario changes completely for the laser-treated samples, both as compared to the thermal-treated ones and in dependence of the dispersion solvent. In general, laser-treated samples present a more complex size and shape distributions, with size reduction of the aggregates as well as re-aggregation. More in detail, size reduction occurs in the H_2_O15, P2OL15, H_2_O44, P2OL44 samples where aggregates of a size down to 20–30 nm are observed, along with larger ones of up to 300 nm (Fig. 4e, for H_2_O15). The most striking feature of H_2_O15 is the rearrangement into extended sheets of up to 50 μm in the longest direction, as in Fig. 4f rod-like texture is visible, as well blocks deposited on the surface (enlarged in the area delimited by the orange square), whereas small aggregates are distributed all around the sheet.

The sheet may fold to create compact 3D structures (Fig. 4g), though small clusters are still visible inside the folds, as it can be observed inside the magnified in Fig. 4h. After 44-min laser irradiation of PG36 in water dispersion, fibrous morphology becomes heavily dominant (Fig. 4i). Large aggregates of smaller roundish particles are present surrounded by a characteristic extended mesh, with a 50 nm pace (Fig. 4j). Two main features attain laser-treated PG36 in propan-2-ol, the presence of fibers with aspect ratio L/D between 8 and 26 (both P2OL15 and P2OL44) and the generation of a beam sensitive foam-like structure, which is uniformly distributed over the whole surface of the sample holder in P2OL15 and becomes droplet-like in P2OL44 (Fig. 4k, l). Additional laser treatments were performed in milder fluence conditions (0.021 J/cm^2^) to investigate to onset of these features (Fig. 4m, n). The foamy texture is more extended and presents embedded aggregates and nanoparticles as can be observed in Fig. 4n. In addition, regularly shaped crystalline-like features appear as in Fig. 4m.

Low fluence laser treatments were performed on the commercial ink Green Concentrate (GConc) based on the pigment PG7 (Bauer et al. 2020) and its extracted part. As compared to GConc treatments, there are some common features, such as simultaneous size reduction of the aggregates and re-aggregation, whereas flower-like structures are absent in the current case. As for fibers, though already observed upon GConc, the presence of fibers upon PG36 lasing is definitively awkward, because of the ascertained presence of SiO_2_. It must be added that, in the current study, fibers are also observed upon laser treatment of PG36 in water and propan-2-ol; whereas, in case of GConc, they were generated only in propan-2-ol for the extracted ink. In general, if phthalocyanines only are deemed as taking part to the re-aggregation process, the lower viscosity solvent favors the fibers formation. However, the presence of siloxanes/SiO_2_ shifts the overall scenario towards fibers formation in water too. Peculiar to PG36 are the foamy structures upon treatments in propan-2-ol only, which form already at low fluence and may embed nanoparticles, thus creating larger conglomerates. These particular structures are beam sensitive (see Fig. SI5), hinting at a large hydrocarbon content (likely linear chains as also pointed by the GC–mass spectra), which may account both for foamy appearance and the reactivity to the beam irradiation.

To assess the hydrodynamic size of laser- and thermal-treated samples, we performed DLS measurements. It must be noted that the samples subjected to thermal treatments are powders which are suspended in solvents for DLS measurements, whereas laser treatments are already performed on dispersions. Extended structures such as those found in Fig. 4f, g are not revealed by DLS because they undergo rapid sedimentation after dispersion, which is a requirement for the measurement to be reliable, since it is based on the intensity fluctuation of a constant number of particles per scattering volume undergoing Brownian diffusion in the suspension. This implies the exclusion of large particles, aggregates or dust.  On the other hand, objects with the size of tenths of nm went undetected in presence of a distribution of large aggregates, since this is dominating, even if large aggregates are a small amount. This is related to the adopted analysis method basing on the intensity-weighted size distribution, where the intensity is weighted according to the scattering intensity of each particle fraction or family, which is proportional to the square of the molecular weight (De Vos et al. 1996).

Results of DLS analyses are reported in Table 6. Laser-treated samples dispersed in propan-2-ol show objects of large size (in the micrometric range) and polydispersity with respect to their analogs dispersed in water. On average, the lower viscosity of propan-2-ol seems to favor the formation of larger aggregates.

**Table 6:**
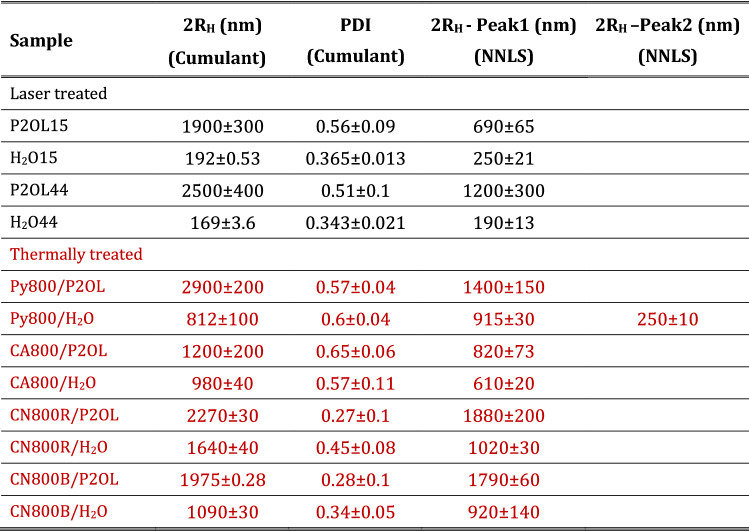
Average hydrodynamic diameter 2*R*_H_ from DLS measurements on PG36 after treatments determined by cumulant analysis and intensity-weighted size distribution by NNLS algorithm

Black fonts correspond to PG36 dispersed in water or propan-2-ol and treated with laser Nd:YAG, the red fonts correspond to thermally treated samples, dispersed afterwards in water or propan-2-ol

As far as the laser-treated samples are concerned two more issues must be taken into account. From one side, siloxanes survive the treatment in water, thus contributing dispersing the products too. On the other hand, the foamy structures in propan-2-ol do contain smaller particles embedded in larger “bubbles”, with an overall large hydrodynamic radius, although the chromophore aggregates are smaller.

The overall comparison of laser and thermal treatments evidences a few issues. From the sheer analysis of the GC–mass spectra, pyrolysis of PG36 appears, in first instance, associated with larger hazards as compared to laser treatments, due to a larger production of harmful fragments compounds, among which there are carcinogenic and organ impairing ones. On the morphological ground, laser treatments appear more dangerous, since they generate nanoparticles and fibers, which are deemed harmful (especially the latter ones). However, the role of the additives must also be taken into account, in association with the on-going processes, i.e., hydrocarbons might ignite a plausible combustion process causing a further fragmentation during the pyrolysis, whereas siloxanes significantly play a role in fiber formation in laser processes. All-in-all, a correct toxicity assessment depends on the actual tattoos’ removal mechanisms. Laser treatments of pigments are often considered as dominated by thermally driven processes, especially if the irradiation wavelength does not correspond to a maximum in the UV–Visible light adsorption of the chromophore.

In a recent paper by Schreiver et al. (2015), a fully photothermal process was hypothesized during laser treatment of the PB15:3 with Ruby laser (694 nm) based on the coincidence of fragment compounds by laser treatment and pyrolysis, also in consideration of the lack of phthalocyanine fragmentation upon irradiation with visible light at the same wavelength. However, when dealing with a fully halogenated phthalocyanine, the scenario changes to some extent, since a large number of fragment compounds can be obtained (for instance halogenated ones), thus revealing possible additional fragmentation mechanisms, besides the thermal ones. The non-coincidence of laser and photothermal processes is further supported by the production of nanoparticles upon laser treatments only, which speaks in favor of a photomechanical process, such as the photoacoustic effect. However, laser induced photo-thermic processes are also active, at least in terms of increase of the dispersion average temperature, which may cause, for instance the *alfa*-to-*beta* phase transition of the phthalocyanines in water dispersion. Under these premises, the tattoo removal process can be considered as a sum of photoacoustic and photothermal effects, and, in first instance, the associated toxicity relates to both processes simultaneously. As for a perspective evolution of the tattoo removal procedure, a suited laser treatment can be envisaged that balances the contribution of photoacoustic and photothermal processes towards the minimization of the toxicity of the tattoos’ removal. It must be added that additives should be screened based not only on their toxicity, but also on the potential harm in connection with the pigments’ removal.

## Conclusions

We comparatively investigated laser and thermal treatments of the phthalocyanine-based green pigment PG36, permitted as tattoo coloring component of tattoo inks in countries where regulations were issued. Ensuing the characterization of the pigment, the treatments were carried out by Nd:YAG laser irradiation of water and propan-2-ol dispersions, pyrolysis at 700 and 800 °C and calcination at 800 °C in air or under N_2_ flow. The outcomes were investigated with various techniques and reveal the presence of additives, such as the harmful cyclic siloxanes and hydrocarbons of several types, which eventually play a role in the removal processes. In particular, we observed a larger amount of different types of harmful halogenated fragment compounds upon pyrolysis, as compared to laser treatments. Simultaneously, cyclic siloxanes survive the pyrolysis and the laser treatment in water, but not laser treatments in propan-2-ol, thus indicating also a role of the solvents (chosen for the different polarities). As for the solid residues of the processes, we made an estimate through the calcination procedure and found that CuO (usually deemed as harmless, unless exceeding critical doses) is the major product containing Cu from the Cu-phthlocyanine. However, if calcinations are carried out in defect of O_2_, additional harmful CuBr, CuCl and possibly Cu_2_O may form as well (Cu_2_OCl_2_ forms too, but the associated hazard is not known). In addition, SiO_2_ is formed as second major component, deriving from the siloxanes decomposition, and whose toxicity is strongly shape dependent, SiO_2_ fibers being a main source of concern. From the morphological point of view, fibers and nanoparticles with size < 20 nm are observed upon laser treatments, along with agglomerates ranging in the hundreds of nanometres to the micrometers range. Additional features, such as foamy structures attain the laser treatments of propan-2-ol dispersions, where the presence of linear chain hydrocarbons may play a role.

Thermal-treated samples present agglomerates in the micrometric range, with tri-dimensional network, in case of pyrolized samples and flatter components for the calcined ones. Laser treatments of pigments are often described as equivalent to thermal treatments (pyrolysis, in particular). However, in the case of halogenated Cu-phthalocyanine, the processes appear to be different both from the point of view of the produced fragment compounds and of the morphology of the products (also in connection with the presence of additives), thus with an effective different type of toxicity, the laser processes pointing at a concurrent photoacoustic effect. This implies that the actual toxicity of the removal process routinely run by laser treatments might be steered as long as the decomposition mechanism can be controlled.

## Supplementary Information

Below is the link to the electronic supplementary material.

Supplementary file1 (DOCX 12630 kb)
