# Supplementary material for: Laser vs. thermal treatments of green pigment PG36: coincidence and toxicity of processes
Source: Arch Toxicol. 2021 May 4;95(7):2367–83. doi: 10.1007/s00204-021-03052-w (PMC8241676; doi:10.1007/s00204-021-03052-w)
Supplement: Supplementary file 1 — Supplementary file1 (DOCX 12630 kb) [file 204_2021_3052_MOESM1_ESM.docx]

**Supplementary Information**

**Laser vs. thermal treatments of green pigment PG36: coincidence and toxicity of processes**

Elvira Maria Bauer^1^, Daniele Cecchetti^2^, Ettore Guerriero^3^, Steven Nisticò^4^, Giulia Germinario^5^, Simona Sennato^6^, Lorenzo Gontrani^7^, Pietro Tagliatesta^2^, Marilena Carbone^2*^

^1^ Italian National Research Council – Institute of Structure of Matter (CNR-ISM), Via Salaria km 23.9, 00015 Monterotondo, RM, Italy

^2^ Department of Chemical Science and Technologies, University of Rome Tor Vergata, Via della Ricerca Scientifica, 1 – 00133 Rome, Italy

^3^ Italian National Research Council – Institute of Atmospheric Pollution Research (CNR-IIA), Via Salaria km 23.9, 00015, Monterotondo, RM, Italy

^4^ Department of Health Sciences, University Magna Graecia, Catanzaro, Italy

^5^ Department of Pure and Applied Science, University of Urbino, Piazza Rinascimento 6, 61029, Urbino, Italy

^6^ Institute of Complex Systems, National Research Council (CNR-ISC), Sapienza Unit, and Physics Department, Sapienza University of Rome, 00185 Rome, Italy

^7^ Department of Chemistry, Sapienza University of Rome, P.le A. Moro 2, Rome, 00185, Italy

^*^corresponding author, email: carbone@uniroma2.it

**Fig. SI1** Calcined PG36 samples: a) in air, b) under N_2_ flow.


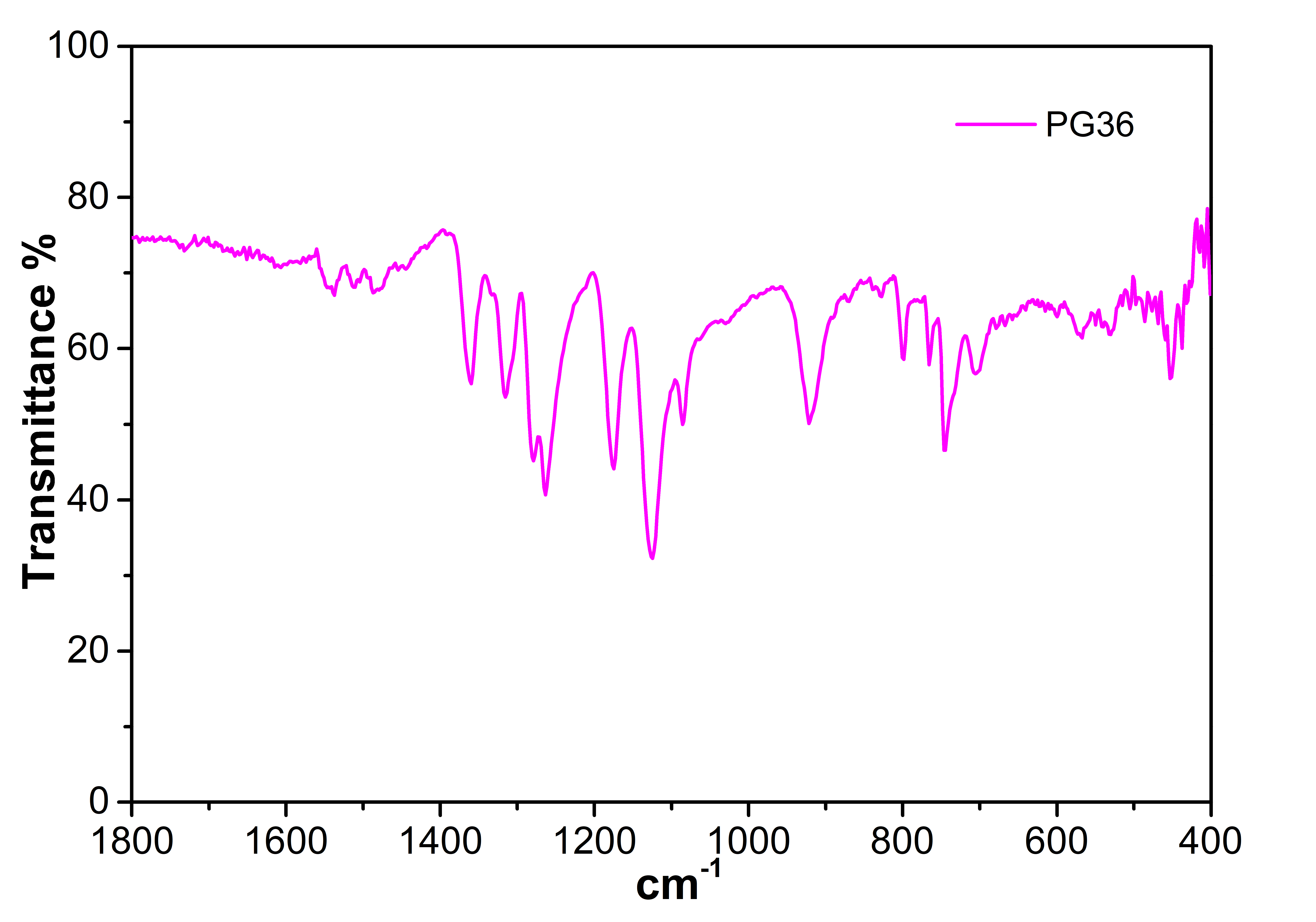


**Fig. SI2** IR Spectrum of PG36.

| RT min | Fragment | Main m/z |
| --- | --- | --- |
| 1.28 | **Carbon dioxide** | 44 |
| 1.37 | **Hydrogen bromide** | 82, 81, 80, 79 |
| 1.37 | **Bromomethane** | 96, 94 |
| 1.51 | **Carbonitridic bromide** | 107, 105, 81, 79 |
| 1.91 | **Bromochloroethene** | 144, 142, 140, 107, 105, 63, 61 |
| 2.02 | **Bromochloromethane** | 132, 130, 128, 95, 93, 91, 51, 49, 47 |
| 2.39 | **Benzene** | 78, 77, 52, 51, 50 |
| 2.78 | **Dibromoethene** | 188, 186, 184, 107, 105, 81, 79 |
| 2.95 | **Dibromomethane** | 176, 174, 172, 95, 93, 81, 79 |
| 3.10 | **Bromodichloromethane** | 165, 163, 161, 131, 129, 127, 85, 83, 49, 47 |
| 3.74 | **Dibromoethene** | 188, 186, 184, 107, 105, 81, 79 |
| 3.98 | **Octamethyl cyclotetrasiloxane (D4)** | 281, 265, 249, 207 |
| 4.27 | **Toluene** | 92, 91 |
| 5.13 | **Dibromochloromethane** | 212, 210, 208, 206, 175, 173, 171, 162, 160, 158, 131, 129, 127, 93, 91, 81, 79, 50, 48, 47 |
| 5.27 | **Bromomethylacetate** | 154, 152, 73 |
| 5.33 | **Dibromoethane** | 190, 188, 186, 109, 107, 95, 93, 81, 79 |
| 5.45 | **Bromobenzene** | 158, 156, 77, 51 |
| 5.52 | **Hexamethyl cyclotrisiloxane (D3)** | 207, 191, 133, 96 |
| 6.13 | **Chlorobenzene** | 114, 112, 77 |
| 6.40 | **Dibromodichloro methane** | 211, 209, 207, 205, 167, 165, 163, 161, 130, 128, 126, 93, 91, 81, 79, 47 |
| 6.63 | **4,5-Dimethyl-4-hexen-3-one** | 126, 97, 69, 57, 41 |
| 6.68 | **1,1-Dibromo-prop-1-ene** | 202, 200, 198, 121, 119 |
| 6.77 | **Dibromochloro ethene** | 224, 222, 220, 218, 143, 141, 139 |
| 7.05 | **Tribromomethane** | 256, 254, 252, 250, 175, 173, 171, 162, 160, 158, 94, 93, 92, 91, 81, 79 |
| 7.31 | **Decamethyl cyclopentasiloxane (D5)** | 281, 265, 249 |
| 7.63 | **2,2-dibromo acetonitrile** | 201, 199, 197, 120, 118, 93, 91, 81, 79 |
| 7.85 | **2,2,4-Trimethyl-hex-3-ene** | 126, 111, 97, 83, 79, 69, 57, 55, 43, 41, 39 |
| 8.13 | **1,4-Dibromobut-2-ene** | 137, 135, 54, 53 |
| 8.50 | **1,1,2-Tribromoethene** | 268, 266, 264, 262, 187, 185, 183, 106, 104, 81, 79 |
| 8.92 | **Benzoic acid** | 122, 105, 77, 51, 50 |
| 9.46 | **1,1,2-Tribromoethane** | 270, 268, 266, 264, 189, 187, 185, 108, 107, 106, 105, 79, 81 |
| 9.11 | **1-Dodecene** | 168, 140, 125, 111, 97, 83, 69, 55, 43, 41 |
| 9.88 | **Tetrabromomethane** | 255, 253, 251, 249, 174, 172, 170, 162, 160, 158, 93, 91, 81, 79 |
| 10.56 | **Dodecamethyl cyclohexasiloxane (D6)** | 429, 341, 324, 207, 147, 73 |
| 10.67 | **Nonanoic acid** | 129, 115, 98, 83, 73, 60, 55, 43, 41 |
| 11.32 | **2,6,11-Trimethyldodecane** | 169, 127, 113, 99, 85, 71, 57, 43, 41 |
| 11.43 | **Dibromobenzene** | 238, 236, 234, 157, 155, 75, 50 |
| 11.43 | **Benzodinitrile** | 128 |
| 11.72 | **Tetrabromoethene** | 348, 346, 344, 342, 340, 267, 265, 263, 261, 186, 184, 182, 172, 105, 103, 93, 91, 81, 79 |
| 12.50 | **Hexadecan-1-ol** | 196, 168, 139, 125, 97, 83, 69, 55, 43, 41 |
| 12.92 | **1,3-Dibromo-5-chloro benzene** | 274, 272, 270, 268, 193, 191, 189, 75, 74 |
| 13.09 | **Tetradecamethyl cycloheptasiloxane (D7)** | 415, 399, 383, 341, 327, 281, 249, 221, 207, 147, 73 |
| 13.21 | **2,6-Dibromophenol** | 254, 252, 250, 173, 171, 145, 143, 63 |
| 13.38 | **4-Bromobenzonitrile** | 183, 181, 102, 50, 51 |
| 13.61 | **3,5-Dibromo-4-hydroxy benzonitrile** | 279, 277, 275 |
| 13.94 | **(1,2-Dibromoethyl) benzene** | 185, 183, 104, 103, 78, 77, 51 |
| 14.29 | **Di-tert-butylphenol** | 206, 191, 57 |
| 14.37 | **1,2,4-Tribromobenzene** | 318, 316, 314, 312, 237, 235, 233, 156, 154, 75, 74 |
| 14.40 | **Heptacosane** | 155, 141, 127, 113, 99, 85, 71, 57, 43, 41 |
| 14.41 | **Tetrabromobutane** | 297, 295, 293, 291, 215, 213, 211, 187, 135, 133, 131, 107, 105, 53, 51 |
| 14.50 | **2-Bromonaphtalene** | 208, 206, 127, 126, 63 |
| 14.91 | **3,4,5-tribromo-1H pyazole** | 308, 306, 304, 302, 227, 225, 223, 200, 198, 196, 146, 144, 93, 91 |
| 15.18 | **Hexadecamethyl cyclooctasiloxane (D8)** | 415, 401, 355, 326, 281, 267, 221, 147, 73 |
| 15.67 | **1,2-Diethylbenzene-1,2-dicarboxylate** | 177, 176, 150, 149, 121, 105, 93, 76, 65 |
| 15.73 | **2,3-Dibromothiophene** | 244, 242, 240, 163, 161, 82 |
| 16.21 | **2,3,4,5-Tetrachloro phenol** | 234, 232, 230 |
| 16.28 | **Bromobenzene-dicarbonitrile** | 208, 206, 127 |
| 16.40 | **Diphenylmetanone** | 182, 105, 77, 51 |
| 16.44 | **2,6,10,15-Tetramethylheptadecane** | 296, 167, 155, 141, 127, 113, 99, 85, 71, 57, 43, 41 |
| 16.61 | **Tetrachlorobenzene- carbonitrile** | 243, 241, 239 |
| 16.69 | **1,4-dibromonaftalene** | 288, 286, 284, 207, 205, 125 |
| 16.70 | **2,6-Bis(propan-2-yl) naphthalene** | 212, 197, 155, 141, 128, 115, 91 |
| 16.77 | **Tetrabromo benzene** | 398, 396, 394, 392, 390, 317, 315, 313, 311, 262, 234, 232, 155, 153 |
| 16.85 | **Dibromochlorobenzene-carbonitrile** | 299, 297, 295, 293 |
| 16.85 | **1,4-dichloro naphthalene** | 200, 198, 196 |
| 16.91 | **Octadecamethyl-cyclononasiloxane (D9)** | 429, 401, 355, 341, 295, 281, 267, 221, 207, 147, 73 |
| 17.07 | **2,6,10,14-Tetramethylhexadecane** | 210, 197, 182, 169, 155, 141, 127, 113, 99, 85, 71, 57, 55, 43, 41 |
| 17.24 | **Tribromophenol** | 334, 332, 330, 328, 253, 251, 249 |
| 17.24 | **2,6-Bis(propan-2-yl) naphtalene** | 212, 197, 155, 141, 128, 115, 91 |
| 17.34 | **Bromo-chlorobenzene-dicarbonitrile** | 244, 242, 240 |
| 17.60 | **10-Heneicosene** | 167, 155, 139, 125, 111, 97, 83, 69, 57, 55, 43, 41 |
| 17.71 | **2-Phenylethyl butanoate** | 104, 91, 71, 43 |
| 18.09 | **Bromochloro benzene- dicarbonitrile** | 244, 242, 240 |
| 18.20 | **1,4-dibromo-2,3,5,6-tetrachlorobenzene** | 380, 378, 376, 374, 372, 370, 299, 297, 295, 293, 291, 218, 216, 214, 212 |
| 18.58 | **Dibromo benzene- dicarbonitrile** | 288, 286, 284, 207, 205, |
| 18.70 | **Didodecyl benzene-1,2-dicarboxylate** | 167, 149, 104, 76, 57, 55 |
| 19.19 | **Dibromodichloro benzene-dicarbonitrile** | 358, 356, 354, 352, 277, 275, 273, 194, 196, 124 |
| 19.23 | **2,4,6-tribromo benzenamine** | 333, 331, 329, 327, 252, 250, 248 |
| 19.30 | **Tetrachlorodimethoxy benzene** | 280, 278, 276, 274 |
| 19.33 | **Tetrabromobenzene** | 398, 396, 394, 392, 390, 317, 315, 313, 311, 236, 234, 232, 154, 152 |
| 19.53 | **n-Hexadecanoic acid** | 256, 213, 185, 171, 157, 143, 129, 115, 111, 97, 83, 73, 69, 57, 55, 43, 41 |
| 19.58 | **3,4,5,6-tetrabromopyridin-2-amine** | 414, 412, 410, 408, 406, 333, 331, 329, 327, 252, 250, 248 |
| 19.92 | **3,4,5,6-Tetrachloro-benzene-1,2-dicarbonitrile** | 270, 268, 266, 264 |
| 20.09 | **Tetrabromothiophene** | 405, 403, 401, 399, 397, 324, 322, 320, 318, 242, 240, 238, 159, 161 |
| 20.35 | **Tribromochloro benzonitrile** | 377, 375, 373, 371 |
| 20.68 | **Tribromochloro benzene-carbonitrile** | 377, 375, 373, 371, 324, 322, 320, 318 |
| 20.68 | **Tribromo thiophene** | 324, 322, 320, 318 |
| 21.20 | **Bromotrichlorobenzene-dicarbonitrile** | 314, 312, 310, 308, 233, 231, 229, 198, 196, 194, 161, 159, 124 |
| 21.46 | **1-Hexacosene** | 364, 135, 153, 139, 125, 111, 97, 83, 69, 57, 55, 43, 41 |
| 21.46 | **1,2,3,4-tetrahydro-1-methyl-7-(1-methylethyl)-phenanthrene** | 238, 223, 207, 195, 181, 167, 165, 153 |
| 21.51 | **Tetrabromo benzene carbonitrile** | 423, 421, 419, 417, 415, 342, 340, 338, 336, 261, 259, 257, 180, 178, 99 |
| 21.73 | **Tribromobenzene-dicarbonitrile** | 368, 366, 364, 362, 287, 285, 283, 206, 204, 125 |
| 23.12 | **Tetracosamethyl-cyclododecasiloxane (D12)** | 429, 415, 401, 369, 355, 341, 327, 325, 295, 281, 267, 221, 207, 147, 73 |
| 23.68 | **Tribromochlorobenzene-dicarbonitrile** | 400, 398, 396, 321, 319, 317 242, 240, 238, 124 |
| 24.33 | **Octacosane** | 365, 350, 322, 308, 294, 281, 266, 253, 239, 225, 211, 197, 183, 169, 155, 141, 127, 113, 99, 85, 71, 57, 43, 41 |
| 24.94 | **Tetrabromo benzodinitrile** | 448, 446, 444, 442, 440, 367, 365, 363, 361, 286, 284, 282, 205, 203, 124 |
| 26.35 | **Hentriacontane** | 197, 183, 169, 155, 141, 127, 113, 111, 99, 97, 85, 71, 57, 43 |
| 27.65 | **Tetratetracontane** | 183, 169, 155, 141, 127, 113, 99, 97, 85, 71, 57, 43, 41 |
| 29.96 | **Tetrabromophthalimide** | 467, 465, 461, 459, 422, 421, 420, 419, 418, 417, 416, 415, 396, 394, 392, 390, 236, 234, 232, 230, 157, 155, 153, 151, 118, 117, 116, 115, 114, 72 |

**Table SI1** Main mass losses of the fragment compounds produced either by pyrolysis or by laser treatments. In case the fragments appear at more than one retention time, it is the fragmentation losses are reported only for the lowest one.

| **H225** Highly flammable liquid and vapour |
| --- |
| **H226** Flammable liquid and vapour |
| **H228** Flammable solid |
| **H300** Fatal if swallowed |
| **H301** Toxic if swallowed |
| **H302** Harmful if swallowed |
| **H304** May be fatal if swallowed and enters airways |
| **H305** May be harmful if swallowed and enters airways |
| **H310** Fatal in contact with skin |
| **H311** Toxic in contact with skin |
| **H312** Harmful in contact with skin |
| **H314** Causes severe skin and eye damage |
| **H315** Causes skin irritation |
| **H317** May cause an allergic skin reaction |
| **H318** Causes serious eye damage |
| **H319** Causes serious eye irritation |
| **H330** Fatal if inhaled |
| **H331** Toxic if inhaled |
| **H332** Harmful if inhaled |
| **H335** May cause respiratory irritation |
| **H336** May cause drowsiness or dizziness |
| **H340** May cause genetic defects (state route of exposure if it is conclusively proven that no other routes of exposure cause the hazard |
| **H341** Suspected of causing genetic defects (state route of exposure if it is conclusively proven that no other routes of exposure cause the hazard) |
| **H350** May cause cancer (state route of exposure if it is conclusively proven that no other routes of exposure cause the hazard) |
| **H351** Suspected of causing cancer (state route of exposure if it is conclusively proven that no other routs of exposure cause the hazard) |
| **H360** may damage fertility or the unborn child (state specific effect if known) (state route of exposure if it is conclusively proven that no other routes of exposure cause the hazard) |
| **H361d** Suspected of damaging the unborn child |
| **H361f** Suspected of damaging fertility |
| **H372** Causes damage to organs (or state all organs affected, if known) through prolonged or repeated exposure (state route of exposure if it is conclusively proven that no other routes of exposure cause the hazard) |
| **H373** May to cause damage to organs (or state all organs affected, if known) through prolonged or repeated exposure (state route if it is conclusively proven that no other routes of exposure cause the hazard) |

**Table SI2** Correspondence between hazard code (reported in red) and toxicity of the fragments generated upon pyrolysis or upon laser treatments.

| RT min | H_2_O Supernatant | P2OL Supernatant |
| --- | --- | --- |
| 3.98 | **D4 Siloxane** |  |
| 6.63 | **4,5-Dimethyl-4-hexen-3-one** |  |
| 7.17 | **Dodecane** |  |
| 7.31 | **D5 Siloxane** |  |
| 7.85 | **2,2,4-Trimethyl-hex-3-ene** |  |
| 9.11 | **1-Dodecene** |  |
| 9.63 |  | 2,2-dimethyl-3-decene |
| 10.56 | **D6 Siloxane** |  |
| 11.32 | **2,6,11-Trimethyldodecane** |  |
| 12.19 | **n-decanoic acid** |  |
| 12.50 | **Hexadecan-1-ol** | Hexadecan-1-ol |
| 12.60 |  | Tetradecane |
| 13.09 | **D7 Siloxane** |  |
| 14.40 | **Heptacosane** |  |
| 15.18 | **D8 Siloxane** |  |
| 15.25 | **Hexadecan-1-ol** | Hexadecan-1-ol |
|  |  | Nonadecane |
| 16.04 | **D8 Siloxane** |  |
| 16.91 | **D9 Siloxane** |  |
| 17.60 |  | 10-Heneicosene |
| 18.44 | **D8 Siloxane** |  |
| 18.70 | **Didodecyl benzene-1,2-dicarboxylate** |  |
| 19.81 | **D8 Siloxane** |  |
| 21.04 | **D9 Siloxane** |  |
| 22.17 | **D9 Siloxane** |  |
| 23.23 | **D12 Siloxane** |  |

**Table SI3** GC-mass spectra of water and propan-2-ol supernatant of PG36. The compounds still present after the laser treatments are reported in light blue, compounds related to fragments generated or freed upon treatment are reported in violet.


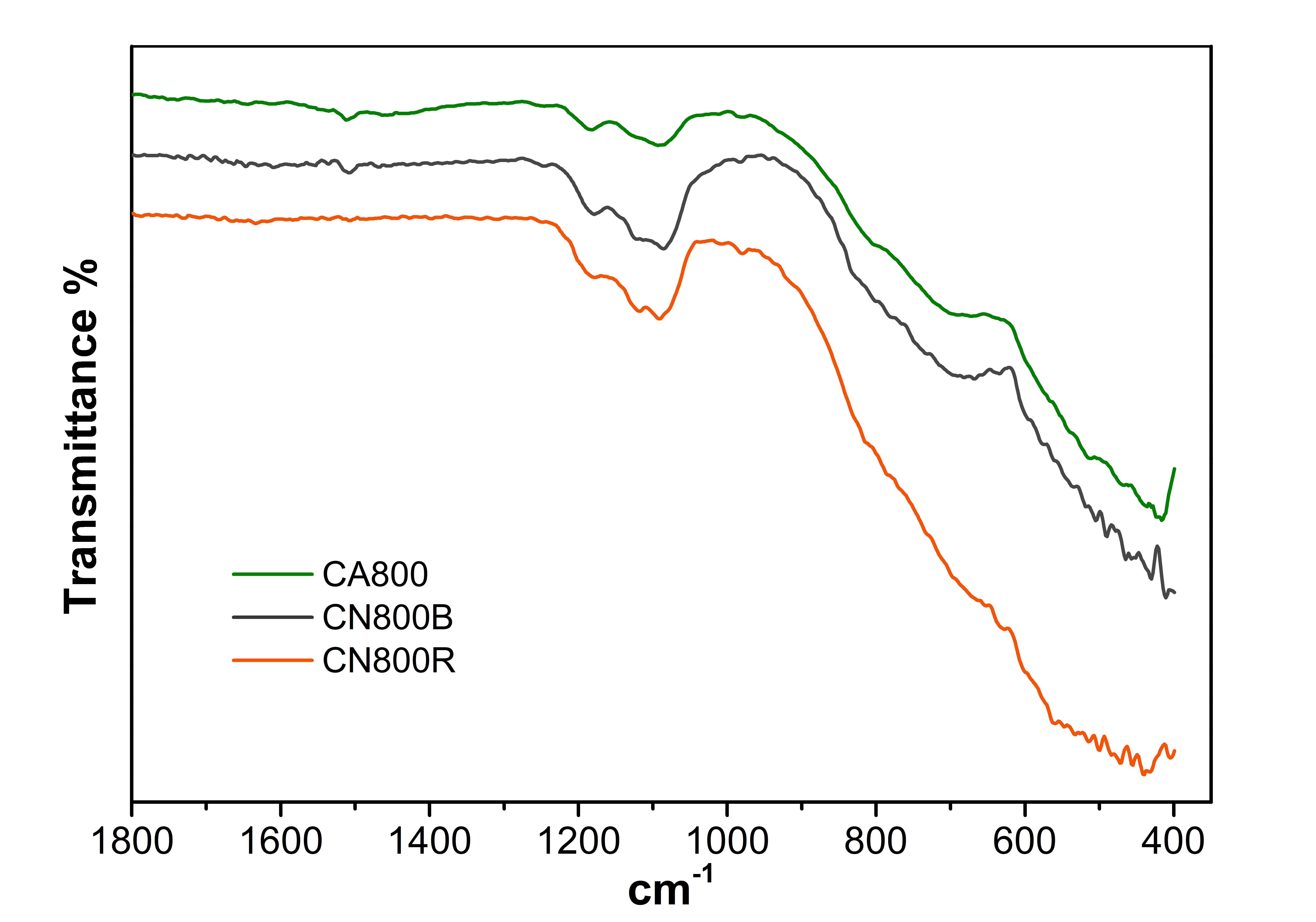


**Fig. SI3** IR spectra of PG36: green solid line (**—**) calcined in air at 800°C, black solid line (**—**) calcined under nitrogen at 800°C, blackish particles, (**—**) light red solid line calcined under nitrogen at 800°C, light red particles.

**Fig. SI4** Comparison between experimental X-Ray powder diffraction pattern of calcined under nitrogen at 800°C, (**—** light red particles) and the theoretical patterns calculated for the following mineral phases (from top to bottom): CuO (|), SiO_2_ (|), CuBr (|), CuCl (|), Cu_2_OCl_2_ (|), Cu_2_O (|).

**Fig SI5** SEM image of low fluence laser treated PG36 in propan-2-ol dispersion, after scanning the area at decreasing magnifications.
